# Extracellular vesicles: cross-organismal RNA trafficking in plants, microbes, and mammalian cells

**DOI:** 10.20517/evcna.2023.10

**Published:** 2023-06-19

**Authors:** Qiang Cai, Lida Halilovic, Ting Shi, Angela Chen, Baoye He, Huaitong Wu, Hailing Jin

**Affiliations:** ^1^State Key Laboratory of Hybrid Rice, College of Life Sciences, Wuhan University, Wuhan 430072, Hubei, China.; ^2^Hubei Hongshan Laboratory, Wuhan 430072, Hubei, China.; ^3^Department of Microbiology and Plant Pathology, Center for Plant Cell Biology, Institute for Integrative Genome Biology, University of California, Riverside, CA 92507, United States.

**Keywords:** Extracellular vesicles, small RNAs, cross-kingdom RNA interference, plant-microbial interaction, spray-induced gene silencing

## Abstract

Extracellular vesicles (EVs) are membrane-enclosed nanometer-scale particles that transport biological materials such as RNAs, proteins, and metabolites. EVs have been discovered in nearly all kingdoms of life as a form of cellular communication across different cells and between interacting organisms. EV research has primarily focused on EV-mediated intra-organismal transport in mammals, which has led to the characterization of a plethora of EV contents from diverse cell types with distinct and impactful physiological effects. In contrast, research into EV-mediated transport in plants has focused on inter-organismal interactions between plants and interacting microbes. However, the overall molecular content and functions of plant and microbial EVs remain largely unknown. Recent studies into the plant-pathogen interface have demonstrated that plants produce and secrete EVs that transport small RNAs into pathogen cells to silence virulence-related genes. Plant-interacting microbes such as bacteria and fungi also secrete EVs which transport proteins, metabolites, and potentially RNAs into plant cells to enhance their virulence. This review will focus on recent advances in EV-mediated communications in plant-pathogen interactions compared to the current state of knowledge of mammalian EV capabilities and highlight the role of EVs in cross-kingdom RNA interference.

## INTRODUCTION

Extracellular vesicles (EVs) were first observed in plants and mammals in the mid-1900s, half a century before it would be known that these vesicles are a universally conserved and impactful form of inter-cellular and inter-organismal communication. The first mammalian observation of extracellular vesicles was made in 1946 when platelet-derived particles were detected in human plasma^[1]^. In the following decades, there were numerous scattered observations of mammalian EVs in vivo and in cell culture^[2]^. However, throughout the 1900s, these extracellular particles were often considered to be cellular debris, a term that historically suggested that these particles represented cellular waste^[3]^. This "debris" designation spurred a decades-long delay in the development of EV research, but since the turn of the century, this field has quickly expanded to show the diversity and influential functions of EVs on cellular development and interactions.

Research on plant- and microbial-derived EVs has undergone the same slow development as the study of mammalian EVs, although in recent years, there has been a surge of new studies characterizing EVs from plant and microbial cells, showing the same scope of diversity and biological significance as mammalian EVs. In cotton plants, intraluminal vesicles in multivesicular bodies were first observed in 1965^[4]^. This was followed by the observation in carrots that multivesicular bodies could fuse with the plasma membrane and release vesicles into the extracellular space, leading to the discovery of plant extracellular vesicles^[5]^. In the early 2,000s, studies of plant and microbial EVs using transmission electron microscopy revealed the accumulation of vesicles at plant-bacteria and plant-fungus interaction sites^[6-8]^. This inspired a new field of research into the role of plant and microbial EVs in the trafficking of materials not only between cells within an organism, but also between different organisms and kingdoms of life.

Numerous classifications and diverse roles of EVs have been characterized in mammalian systems. EVs can be isolated from plasma, serum, urine, and various other physiological fluids^[9,10]^. They are small lipid-encapsulated nanoparticles that carry cargoes, such as RNAs [e.g., small RNAs (sRNAs), messenger RNAs (mRNAs), transfer RNA (tRNA), and long non-coding RNAs], DNA fragments, lipids, and proteins^[9,11-13]^. EVs transport these biomolecules between cells and play an important role in cell-to-cell communication^[9,14]^. In addition, EVs have emerged as important disease biomarkers and have potential applications in therapeutics and diagnostics^[15-17]^. Among all cellular origins, including mammalian, plant, and microbial cells, EVs can be broadly classified into either exosomes or microvesicles based on their biogenesis^[3,10,18]^. Exosomes are small EVs (30-150 nm in diameter) originating from the endosomal organelle multivesicular bodies (MVBs). Microvesicles, also denoted as ectosomes, are heterogeneous and larger EVs (100-1,000 nm in diameter) generated by direct budding from the plasma membrane^[19,20]^. In contrast to exosomes and microvesicles, mammalian cells also produce several types of vesicle bodies associated with different forms of cell death^[3]^. These include necroptotic vesicles released from necroptotic cells^[21,22]^, pyroptotic inflammasomes released during pyroptosis of cells^[23]^, and apoptotic bodies (1-5 µm in diameter) produced during apoptotic cell disassembly^[24-26]^. Research on EVs from plants and microbes has progressed less than research in mammalian systems due to the limitations in EV isolation and detection methods^[27]^. Mammalian EVs are frequently isolated from cell cultures or body fluids such as blood, breast milk, urine, lung fluid, and semen^[2,28]^. Since these mammalian samples are fluids, extraction is relatively straightforward. In contrast, extraction of EVs from plants is more challenging due to the small amount of extracellular fluid and the extensive procedures required for EV isolation in plants.

**Figure 1:**
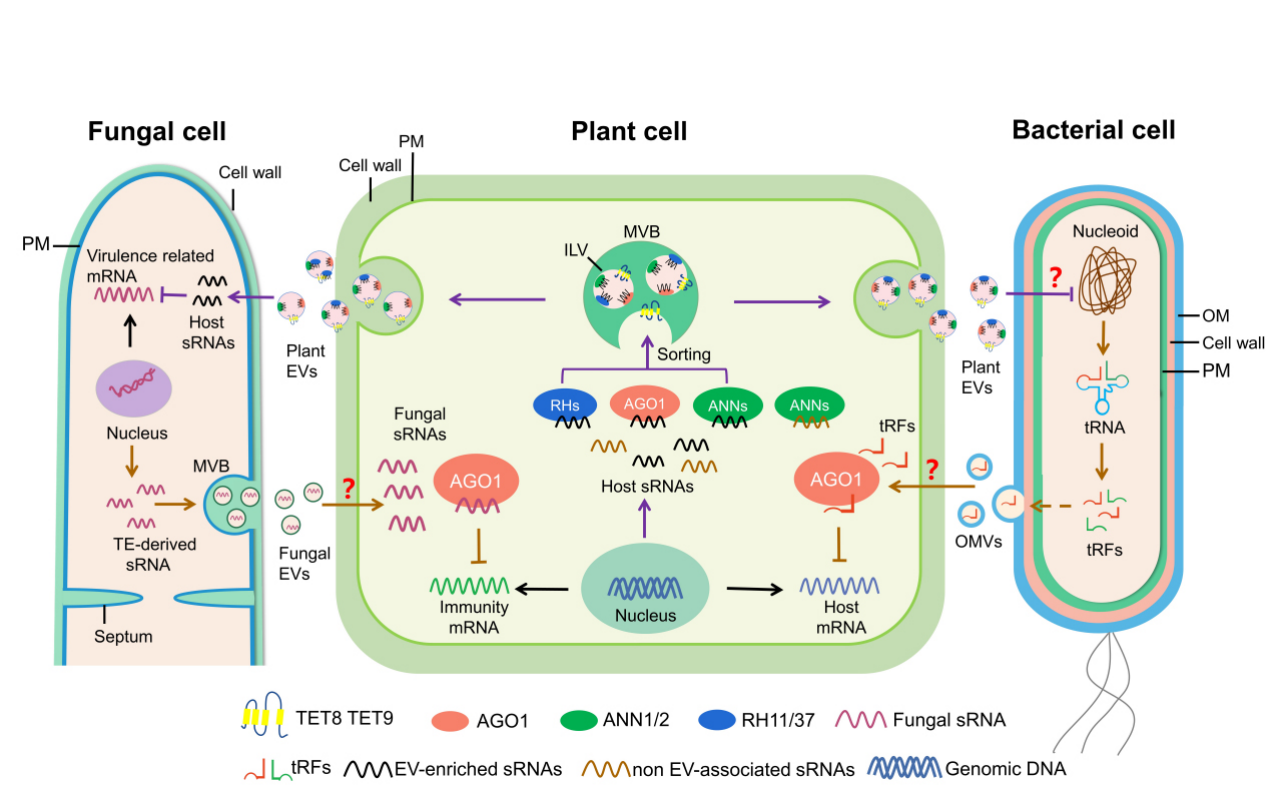
Cross-kingdom RNAi in plant–microbial interactions. Some fungal pathogens, such as *B. cinerea* and *V. dahlia*, deliver sRNAs into the plant cells to silence host genes that are involved in plant immunity^[88,91]^. Cross-kingdom RNAi is bidirectional, and plants secrete TET8/9-positive EVs to transport host functional sRNAs into pathogens to silence fungal genes involved in virulence^[29]^. TET8/9-positive EVs contain a variety of RBPs, including AGO1, RHs, and ANNs, which contribute to the selection or stabilization of sRNAs in EVs^[56]^. Cross-kingdom RNAi also exists in bacteria-plant interaction. Rhizobia tRNA-derived short fragments act as functional sRNAs moving into plant cells to silence target genes related to nodulation^[113]^. sRNAs from fungal pathogen and bacterium rhizobia were all found to be loaded into plant host AGO1 to silence host target genes. Fungi and bacteria are predicted to secrete and transport sRNAs into host cells by EVs. The question mark indicates a prediction that has not yet been validated experimentally. EVs: extracellular vesicles; MVB: multivesicular body; ILV: intraluminal vesicle; OM: Outer membrane; PM: plasma membrane; sRNA: small RNA; TE: Transposable element; tRFs: tRNA-derived fragments.

Recently, plant EVs have been successfully isolated from the apoplastic fluids of leaves, roots, and seeds^[29-34]^. Research has attempted to characterize different classes of plant EVs, protein markers, and lipid composition, mirroring the work done to characterize mammalian EVs. Emerging evidence indicates that plant EVs play an essential role in the cross-kingdom transport of plant sRNA into fungal cells to silence virulence-related genes^[29,35,36]^. This phenomenon, termed "cross-kingdom RNAi", has been observed in interactions between numerous plant and animal hosts with their associated microbes and parasites^[35,37-41]^. In some host-pathogen/parasite interactions, RNA translocation has been linked to EV trafficking^[29,42-46]^. The wide range of organisms capable of RNA exchange highlights the importance of research into the mechanisms of RNA translocation and the potential role of EVs in these interactions. Recent research has also shown that for plant-infecting microbes such as bacteria, fungi, and oomycetes, EVs could play a prominent role in pathogenesis by delivering proteins, RNAs, and metabolites into their plant host cells^[47-52]^ [Figure 1]. In this review, we highlight and discuss the current state of research on plant-derived EVs and cross-kingdom RNAi, with an emphasis on the role of EVs in cross-kingdom RNA trafficking. We also discuss plant-interacting pathogen-derived EVs and their biological functions. Finally, we include the potential applications of plant EVs for agricultural crop protection and advances in human medicine.

## PLANT EXTRACELLULAR VESICLES

**Figure 2:**
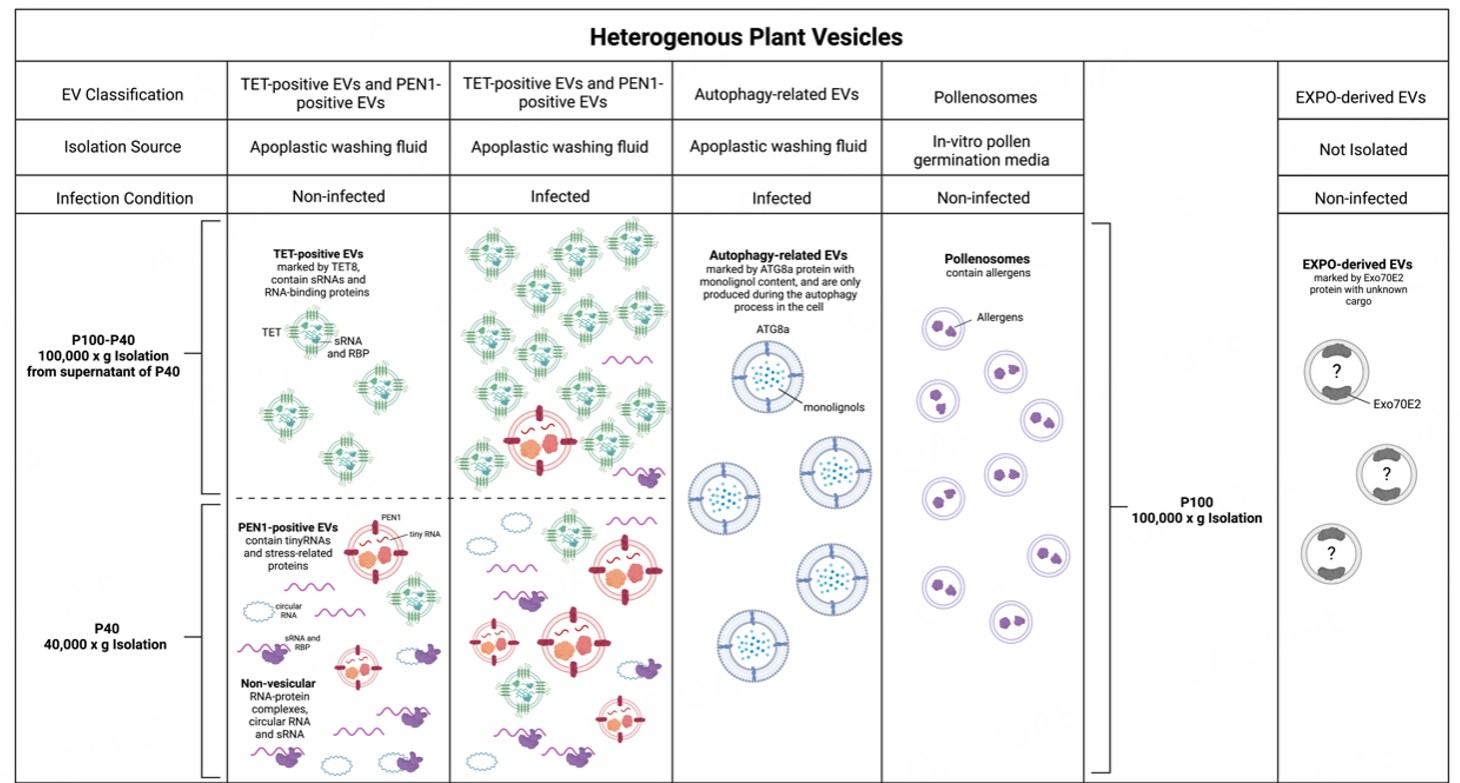
Heterogeneous populations of EVs isolated from plants. There are at least four known EV populations that have been isolated from plants: TET-positive EVs, PEN1-positive EVs, autophagy-related EVs, and pollenosomes. They have different sizes, densities, cargoes, and intracellular origins. Pathogen infection induces secretion of both TET-positive EVs and PEN1-positive EVs^[56]^. In the process of EV isolation, final centrifugation of 40,000 × *g* (P40) pellets larger and heavier vesicles such as PEN1-positive EVs and large EVs, non-vesicular free RNA, and RNA-protein complexes^[30,79]^. Small EVs, such as TET-positive EVs, are mainly present in the supernatant after 40,000 × *g* centrifugation and require a higher speed of ultracentrifugation at 100,000 × *g* for collection (P100)^[30,56]^. Autophagy-related EVs marked with ATG8a were collected using 100,000 × *g* from plants during the autophagy process within cells^[64]^. Pollenosomes secreted during pollen germination and pollen tube growth were collected at 100,000 × *g* from in-vitro pollen gemination media^[59]^. EXPO-derived EVs originate from the plant-specific organelle EXPO, are marked by the protein Exo70E2, and have not yet been isolated from plants^[71]^. This figure was created with https://www.biorender.com/.

EVs provide protection for their biological cargo from the abundant nucleases and proteases within the extracellular environment^[53,54]^. EV-mediated trafficking is one of the major pathways for RNA transport. Mammalian EVs can be isolated from the extracellular environment of biological fluids or culture supernatants^[9,10]^. Similarly, plant EVs from various sources can be isolated from extracellular apoplastic washing fluid and cell culture medium^[29,30]^ [Figure 2]. Plant EVs have been observed in numerous plant species such as cotton^[4]^, carrot^[5]^, and rice^[55]^ and have been isolated from *Arabidopsis thaliana*^[29-31,56]^, sunflower^[32,57]^, olive^[58,59]^, and *Nicotiana benthamiana*^[56,60]^ [Figure 2].

Heterogeneous EV groups have been isolated in plants, which may perform different functions^[61,62]^. In Arabidopsis, five EV subtypes have been reported that are either isolated and/or characterized by different protein markers, sizes and/or biogenesis origins, as described in Figure 2. These subtypes include tetraspanin (TET)-positive EVs^[29,56]^, penetration 1 (PEN1)-positive EVs^[63]^, exocyst-positive organelle (EXPO)-derived EVs^[45]^, autophagy-related EVs^[64]^, and pollensomes^[59]^. TET-positive EVs are the class of plant EVs derived from MVBs and most similar to mammalian exosomes. In Arabidopsis, the secretion of TET8-positive EVs increases after the infection of fungal pathogen *Botrytis cinerea*^[29,56]^. These plant exosomes are enriched in the supernatant after centrifugation of leaf apoplast fluid at 40,000 × *g* (P40 fraction), and can be collected by centrifugation at 100,000 × *g* (P100-P40), the same speed used to collect mammalian exosomes. Plant exosomes are mainly responsible for transporting sRNAs, including microRNAs (miRNAs) and small interfering RNAs (siRNAs), from plants to fungal pathogens to induce cross-kingdom RNAi^[29,56]^. TET8 is a homolog of the mammalian EV-enriched tetraspanin proteins CD9, CD63, and CD81. The CD9-, CD63-, and CD81-labeled mammalian EVs are generated from MVBs^[65]^. In mammalian cells, Rab GTPases are essential for intracellular vesicle movement and strongly influence MVB morphology^[9]^. TET8-labeled plant EVs colocalize with the Arabidopsis MVB-marker Rab5-like GTPase ARA6 and accumulate at fungal infection sites, suggesting that TET8-positive EVs are likely originated from MVB trafficking^[29,56]^. Similarities between plant and mammalian exosomes suggest a conserved mechanism in exosome biogenesis between mammalian and plant cells. In mammals, ceramide plays an important role in exosome biogenesis and release^[66]^. Interestingly, sphingolipids in Arabidopsis EVs are nearly pure glycosyl inositol phosphoryl ceramides (GIPCs)^[67]^. GIPCs are only produced by plant and fungal species and are not present in mammalian EVs^[68]^. The amount of cellular GIPCs is lower in the *tet8* knockout mutant than in the wild type, and the *tet8* mutant secretes fewer EVs^[67]^. Exogenous application of GIPCs can rescue the EV secretion defects in the *tet8* mutant^[67]^, illustrating the important role of TET and GIPCs in plant EV formation.

Less is known about the additional types of plant EVs, such as PEN1-positive EVs, EXPO-derived EVs, and autophagy-related EVs. PEN1 is a plant-specific plasma membrane-associated syntaxin protein^[69]^. The biogenesis pathway of PEN1-positive EVs is currently unclear. PEN1-positive EVs are mostly enriched in the P40 fraction and can be collected by centrifugation of apoplastic fluid at 40,000 × *g*. They mainly carry tiny RNAs with 10-17 nt in length^[63]^. The potential destination and biological function of these tiny RNAs remain to be investigated. EXPO-derived EVs originate from the plant-specific novel organelle EXPO, which is marked in plants by the exocyst protein Exo70E2^[70,71]^. EXPO is independent of endosomes and autophagosomes^[71]^. EXPO-derived EVs are a class of large EVs with diameters ranging from 200-500 nm, larger than exosomes^[70,71]^. To date, EXPO-derived EVs have not been isolated and their specific cargoes and biological functions also remain unclear. Autophagy-related vesicles have recently been shown to be released as EVs at the late stage of pathogen infection^[64]^. Within cells, these autophagosomes colocalize with monolignol metabolites and are implicated in the transport of monolignols across the plant plasma membrane into the apoplast to facilitate lignin deposition as part of the plant defense response to pathogens^[64]^. Similar to mammalian systems, plant EVs have been isolated from various tissue types throughout the organism^[45]^. Small plant EVs were isolated from the media used for in vitro pollen germination and pollen tube growth^[58,59]^. These small EVs are termed pollensomes, and their size ranges from 28 to 60 nm in diameter^[59]^. In addition to EVs being a conserved structure among different mammalian species, EVs are currently being discovered in many plant species. Rice EVs have recently been observed in the periarbuscular space after inoculation with the symbiotic arbuscular mycorrhizal fungi Rhizophagus irregularis or Gigaspora rosea using transmission electron microscopy^[55]^. Whether these EVs carry RNA requires further study.

It is worth noting that not all plant-derived vesicles are EVs. EVs are specifically and naturally secreted vesicles found in the extracellular space of plants and have distinct biomarkers and cellular functions^[30,45]^. Previously, vesicles collected from disrupted or homogenized tissues of grape, broccoli, ginger, and grapefruit were termed nanovesicles^[72,73]^. However, these nanovesicles are not true EVs, because they do not naturally occur in extracellular spaces. Nevertheless, plant nanovesicles have biomedical applications because they can protect and deliver biological compounds, such as drugs, RNAs and proteins, into target cells^[74]^. Plant nanovesicles play a role in the prevention of inflammation and intestinal permeability in humans and mice and influence the intestinal microbiome^[75]^. Consistent with their separate classifications, plant nanovesicles and Arabidopsis-derived EVs have different size distributions: EVs range from 60-200 nm, and nanovesicles range from 100-300 nm^[76]^. Cancer cells take up both types of vesicles with high efficiency, although EVs have an almost three-fold higher uptake rate than plant nanovesicles^[76]^. This result highlights the functional diversity of plant-derived vesicles and supports the idea that plant EVs have the natural property of transporting cargo between cells. These results suggest that plant EVs have potential applications in the delivery of therapeutics^[77,78]^.

## COMMONLY USED METHODS TO EXTRACT PLANT EXTRACELLULAR VESICLES

For mammalian cells, EVs can be isolated from extracellular fluids using differential centrifugation to obtain different subgroups of EVs with different density ranges^[9]^. For instance, low-speed centrifugation (around 2,000 × *g*) can effectively recover large vesicles or cell fragments like apoptotic bodies. Intermediate-size EVs, such as microvesicles, can be collected by centrifuging at around 10,000-20,000 × *g*. Small EVs, predominantly exosomes, require high-speed ultracentrifugation over 100,000 × *g* (P100) for successful recovery. In plants, two ultracentrifugation speeds have been used to pellet EVs: 40,000 × *g*, which collects the P40 fraction, and 100,000 × *g*, which collects the P100 fraction. For plant EVs pelleted at 100,000 × *g*, 92% have diameters in the range of 30-150 nm^[30]^. Mammalian EVs pelleted at the same ultracentrifugation speed have similar diameters below 150 nm and are denoted as exosomes^[9,10,61]^. In plants, the P100 fraction is enriched in the exosome-like TET8-positive EVs, which contain sRNAs and RNA-binding proteins^[79]^. Most TET8-positive EVs remain in the P40 supernatant, and centrifugation of the supernatant of P40 at 100,000 × *g* (the P100-P40 fraction) collects mostly TET8-positive EVs^[79]^ [Figure 2]. The P40 fraction is enriched in PEN1-positive EVs, which contain tiny RNAs and proteins^[79]^. As well in the P40 fraction, non-vesicular sRNAs and circular RNAs associated with RNA binding proteins co-pellet with EVs^[79]^. While circular RNAs are more resistant to degradation because of lacking free ends, it is currently unknown how other liner non-vesicular RNAs, including sRNAs, could be protected from degradation within the plant apoplast that contains numerous nucleases^[53,54]^, and whether RNA binding protein alone can completely protect the non-vesicular RNAs from degradation. These studies using different ultracentrifugation speeds for plant EV isolation are complementary to the discovery and characterization of distinct classes of EVs with different sizes, densities, and cargoes.

In tandem with different centrifugation speeds, differences in the extraction methods of apoplastic washing fluid can greatly affect the collection of different EVs or non-vesicular contents. Studies characterizing the P40 fraction notably isolated EVs and non-vesicular RNAs/RNA-protein complexes from apoplastic washing fluid extracted from the whole plant, rather than detached leaves^[31,79]^. Apoplastic washing fluid collected by the whole plant method contains more contamination from cell debris and cytoplasmic content compared to the extraction of apoplastic washing fluid from the detached leaves method^[30]^. This is evident through Western blotting analysis, which detected a high amount of Rubisco protein in EV fractions isolated with the whole plant method^[30]^. In comparison, the detached leaves method resulted in minimal Rubisco protein contamination in EV preparations^[30]^. It is important to consider not only extraction methods, but also the disease or stress conditions of the plant that affect EV cargoes as well as non-vesicular contents that co-pellet with EVs. Different methods may only capture different subsets of EVs, and it is important to avoid generalizations about the function of all plant EV subtypes based on individual methods.

Differential centrifugation followed by gradient centrifugation can help increase the quality of isolated EVs and reduce contaminations^[29,30]^. Gradient centrifugation can be done using a sucrose gradient^[80]^ or an iodixanol gradient^[31]^. Different plant EV subtypes are found in different density fractions, which has been shown through the detection of EV protein markers^[30,31]^. PEN1-positive EVs are enriched in the iodixanol gradient fraction from 1.029 to 1.056 g/mL^[31]^. TET8-positive EVs are enriched in the iodixanol fraction of 1.08 g/mL^[30]^, which is similar in density to mammalian exosomes (1.08-1.12 g/mL)^[81,82]^. Importantly, TET8-positive EVs are in the same fractions of plant miRNAs and siRNAs^[56]^, suggesting that TET8-positive EVs are the major class of EVs for sRNA transport. Along with pelleting at different final ultracentrifugation speeds, enrichment in different gradient fractions further suggests that TET8‐positive EVs and PEN1‐positive EVs represent two distinct subpopulations with different densities. Furthermore, in transgenic plants co-expressing fluorescently labeled TET8 and PEN1, TET8-GFP-labeled EVs and PEN1-mCherry-labeled EVs do not colocalize, supporting the distinction between these two classes of EVs^[30,56]^. Differential centrifugation, sucrose gradients, and buoyant density gradients also reduce the occurrence of apoptotic cell fragments in the final pellet^[83]^.

Methods for isolation of plant EVs, such as differential centrifugation and gradient density centrifugation, can fractionate different classes of EVs based on size and density, but these methods cannot sufficiently purify one specific EV subtype in isolation. The method that is able to purify one specific EV class is immunoaffinity purification using an antibody that recognizes a specific protein marker of the particular EV class. A TET8 native antibody that recognizes an extracellular domain 2 (EC2) of TET8 has been successfully generated to isolate plant TET8-positive EVs^[30,56]^. Plant miRNAs and siRNAs were detected in immunoaffinity purified TET8-positive EVs^[56]^, supporting that plant sRNAs are mainly transported by exosomes. PEN1-positive EVs and EXPO-derived EVs have not been successfully separated from heterogeneous EV classes. The preparation of antibodies that recognize the extracellular domains of PEN1 and EXO70E2 to purify PEN1-positive EVs and EXPO-derived EVs will help determine the RNA cargoes and other molecules in these subclasses of EVs. It is highly desirable that immunoaffinity capture be used to properly assess specific cargo of different vesicle classes. Recent technical advances in flow field-flow fractionation chromatography may make it possible to sort different classes of EVs by different sizes or fluorescence labeling^[84]^.

## CROSS-KINGDOM RNAI

sRNAs are short non-coding regulatory RNAs that silence genes with complementary sequences^[37,85]^. In eukaryotes, sRNAs are generated by the ribonuclease III-like enzyme Dicer or Dicer-like (DCL) proteins. sRNAs are then incorporated into Argonaute (AGO) proteins to form an RNA-induced silencing complex, which performs gene silencing in a sequence-specific manner by mRNA cleavage and degradation, translational inhibition, or transcriptional gene silencing. During microbial infection, host RNAi machinery is critical for reprogramming gene expression to induce plant immunity^[37,86,87]^. Recent studies demonstrate that, in addition to their endogenous functions, sRNAs travel between hosts and their interacting organisms and induce ‘cross-kingdom or cross-organismal RNAi’ *in trans*^[35,38,88-90]^ [Figure 1].

Cross-kingdom RNAi was first observed in the interaction between Arabidopsis and the pathogen *Botrytis cinerea*^[91]^. During infection on both Arabidopsis and tomato host plants, *B. cinerea* delivers sRNAs into plant cells that silence plant immunity genes by binding to the host AGO1 protein^[91,92]^. Pathogen sRNAs associate with host AGO proteins and target genes involved in plant immune responses, including plant kinase genes, which are often involved in signal transduction pathways that mediate plant defense ^[93]^. *B. cinerea* sRNAs specifically target mitogen-activated protein kinase (MAPK) pathway genes and cell wall-associated kinases^[91]^. Like *B. cinerea*, sRNAs from the fungal wilt pathogen *Verticillium dahliae* also bind to Arabidopsis AGO1 for host gene silencing^[88,94]^. In the fungal rust pathogen of wheat, *Puccinia striiformis*, a micro-like RNA (milRNA) serves as an important pathogenicity factor that silences *pathogenesis-related 2* (*pr2*) gene, encoding a β-1,3-glucanase, which is a key wheat immunity gene^[95]^. As well, other *P. striiformis* sRNAs are predicted to target wheat kinase genes^[96]^. On tomato hosts, the fungal pathogen *Fusarium oxysporum* generates mil-R1, which is loaded into tomato AGO4 to silence the tomato CBL‐interacting protein kinase gene^[97]^. In addition to fungal pathogens, the oomycete pathogen *Hyaloperonospora arabidopsidis* sends sRNAs into Arabidopsis cells that associate with Arabidopsis AGO1 to silence defense-related genes: *with no lysine kinase* (AtWNK)2 and *apoplastic*, *enhanced disease susceptibility-dependent* (AtAED)3^[98]^. sRNAs from the biotrophic powdery mildew pathogen, *Blumeria hordei,* have also been predicted to target possible barley host immunity-related genes^[99]^.

Fungal sRNA effectors play an important role in fungal pathogenicity and virulence. The majority of fungal sRNA effectors are derived from long terminal repeat (LTR) retrotransposons and are generated by fungal DCLs (DCL1 and DCL2)^[91]^. Mutation or silencing of fungal DCL genes can attenuate the virulence and growth of fungal pathogens. Mutation of both DCL genes reduced the virulence and growth of the grey mold pathogen *B. cinerea*^[88,91,100]^, the soilborne pathogen *Fusarium graminearum*^[101]^, the post-harvest decay pathogen *Penicillium italicum*^[102]^, the apple canker pathogen *Valsa mali*^[103]^, and the grapevine downy mildew pathogen *Plasmopara viticola* ^[104]^. Furthermore, even silencing of DCL genes, reducing expression of both DCL genes without knocking out expression, resulted in reduced pathogenicity of *B. cinerea*^[88,100,105]^, *F. graminerum*^[106]^, and *P. viticola*^[104]^. Mutation of DCL genes has also shown reduced virulence and growth rate of the anthracnose pathogen *Colletotrichum gloeosporioide*s^[107]^. TEs are major components of eukaryotic genomes and give rise to many siRNAs^[89,108]^. In phytopathogens, the LTR retrotransposons are associated with virulence in host-adapted subpopulations of *B. cinerea*^[109]^. This phenomenon may be due to sRNA effectors being generated from these LTR regions. Thus, cross-kingdom transported sRNAs originating from these TEs could explain the rapid variation of sRNA species produced during the co-evolutionary arms race with the host.

Beyond fungal pathogens and plants, cross-kingdom RNAi is a conserved mechanism across many diverse species. The parasitic plant dodder *Cuscuta campestris* delivers 22-nucleotide miRNAs into Arabidopsis, which can act as virulence factors during parasitism^[110]^. Cross-kingdom RNA trafficking also occurs in host-symbiontic interactions. For example, to stabilize a symbiotic interaction, the ectomycorrhizal fungus *Pisolithus microcarpus* transports miRNAs into *Eucalyptus grandis* cells to silence host NB-ARC domain-containing genes. This attenuates the superfluous immune response of the plant to accommodate the symbiont^[111]^. As well, in the symbiosis between the arbuscular mycorrhizal fungi *Rhizophagus irregularis* and its host plant *Medicago truncatula*, fungal sRNAs are predicted to target host mRNAs of genes whose expression is modulated in roots after mycorrhizal colonization^[112]^. Furthermore, sRNA trafficking between hosts and microbes is not limited to eukaryotes with RNAi machinery. The plant symbiotic bacterium *Rhizobium* transports 21-nucleotide tRNA-derived sRNA fragments (tRFs) into its soybean host, which are loaded into soybean AGO1 and cleave nodulation-related mRNAs, contributing to the symbiotic interaction^[113]^. It remains to be determined whether cross-kingdom transport of tRFs into plant hosts occurs during pathogenic bacterial infections.

Cross-kingdom RNAi is bidirectional, in which sRNAs are sent from the microbe into the host and also from host cells into interacting microbes. As part of the plant defense response, some plant sRNAs, generated by DCL proteins^[114]^, are transported into interacting microbes/pests and silence target genes necessary for pathogen/pest infection, thereby reducing plant disease^[38]^. In Arabidopsis, trans-acting small interfering RNAs (tasiRNAs) are trafficked into interacting *B. cinerea* cells and silence fungal vesicle trafficking-related genes essential for virulence, such as v*acuolar protein sorting 51* (*VPS51*), *dynactin* (*DCTN1*) and *suppressor of actin*(*SAC1*)^[29]^. The transport of host plant endogenous sRNAs into pathogens has been observed in other pathosystems. Tomato plants transport multiple miRNAs into *B. cinerea* cells that attenuate virulence by suppressing spore germination and silencing virulence-related genes^[115,116]^. Cotton plants produce miR159 and miR166 that respectively target genes in *V. dahlia* that are essential for fungal virulence^[117]^. As well, wheat plants produce a miRNA that silences the expression of a hydrolase enzyme in the fungus *Fusarium graminearum* which directly decreases its virulence^[118]^.

During the arms race between hosts and pathogens, some pathogens have also developed countermeasures to suppress cross-kingdom RNAi. A recent study showed that the fungal pathogen *V. dahliae* has an adaptive mechanism to evade host-derived cross-kingdom RNAi. *V. dahliae* secretes protein secretory silencing repressor 1 (VdSSR1) into plant cells, which interferes with host AGO1-miRNA export from the nucleus and inhibits antifungal cross-kingdom RNAi^[119]^. In addition,to promote infection, the oomycete *Phytophthora capsici* produces an effector called Phytophthora suppressor of RNA silencing 2 (PSR2), which inhibits the biogenesis of Arabidopsis secondary siRNAs that target *Phytophthora* genes via cross-kingdom RNAi^[43]^.

Beyond plant-microbial/parasite interactions, cross-kingdom RNAi or cross-organismal RNAi has also been observed between mammalian and insect hosts and their pathogens/parasites. In insects, the fungal pathogen *Beauveria bassiana* transports a milRNA into mosquito cells and uses the same mechanism as the plant fungal pathogen by hijacking mosquito host AGO1 to silence the expression of a mosquito Toll receptor ligand, thereby attenuating mosquito immunity^[120]^. Conversely, in this interaction, *B. bassiana* infection induces the expression of two mosquito miRNAs, let-7 and miR-100. These miRNAs move into the fungal cells and specifically silence the *sec2p* and *C6TF* fungal genes, both of which are essential for fungal growth and pathogenicity^[121]^. In mammals, the gastrointestinal nematode *Heligmosomoides polygyrus* transports miRNAs into mouse intestinal epithelial cells to suppress innate immune responses and eosinophilia^[42]^. As well, human monocyte cells export miRNAs into the fungal pathogen *Candida albicans* to silence the cyclin-dependent kinase inhibitor *Sol1*^[122]^. Human and mouse gut epithelial cells also secrete miRNAs that can regulate gene expression and growth of gut bacteria, such as *Fusobacterium nucleatum* and *Escherichia coli*^[44]^. Although bacteria do not possess RNAi machinery, bacterial endoribonucleases may mediate this cross-kingdom gene regulation. Alternatively, host sRNAs could be transported together with host AGO proteins to silence bacterial genes *in trans*. There have also been reports about the possible functions of dietary sRNAs and their potential role in modulating endogenous gene expression in mammals. Although different studies using different systems with varying experimental conditions have produced contradictory results, this nascent area of research into the therapeutic effects of dietary sRNAs is worth pursuing^[123]^. These observations suggest that cross-kingdom RNAi occurs throughout many branches of life between various parasites/microbes and their hosts.

## EXTRACELLULAR VESICLES IN CROSS-KINGDOM SMALL RNA TRAFFICKING

Mammalian exosomes from different cell types have been found to carry different RNAs, such as miRNAs, mRNAs, and long non-coding RNAs, throughout the body for various functions in disease, cancer, and immune responses^[3,17]^. For example, human breast cancer cells produce EVs containing miRNA precursors and DICER and AGO2 proteins that function together to perform gene silencing in naive non-cancerous cells^[124]^. Human T-cells also produce exosomes with specific exosome-enriched miRNAs that are transported into infected antigen-presenting cells to modulate their gene expression as part of the immune response^[125]^. Renal cancer cells secrete EVs loaded with a long non-coding RNA that acts in tandem with miRNAs in cells to modulate gene expression and overcome the effects of anticancer drugs^[126]^. Cancer-derived EVs carrying intact mRNAs also facilitate the spread of ovarian cancer into the peritoneal cavity^[127]^. There is a much greater currently characterized diversity of types and functions of RNAs in mammalian EVs compared to plant or microbial EVs.

EVs can be isolated from uninfected healthy plants^[29,31]^ but are more enriched in plants after pathogen infection or treatment with the plant defense hormone salicylic acid^[29,31,56]^, suggesting that EVs play an important role in plant immunity. Comparative analysis on sRNA profiles of purified fungal cells and EVs isolated from the infected tissue show that more than 70% of transferred plant sRNAs found in fungal cells could be detected in plant EVs pelleted at 100,000 × *g*^[29]^. This suggests that EVs are one of the major pathways for cross-kingdom sRNA trafficking. Using density gradient analysis and direct immunoaffinity capture, EV-enriched sRNAs are present in the fractions of TET8-positive EVs^[56]^. Furthermore, double mutants of *Arabidopsis tet*8 and its close homolog *tet9* (*tet8*/*tet9*) deliver much fewer sRNAs into fungal cells and show increased susceptibility to *B. cinerea* infection^[29]^. These biochemical and genetic results further support that TET8-positive EVs are the major class of EVs that transport sRNAs to fungal cells [Figure 1]^[29,56]^. Similarly, Arabidopsis sends phasing siRNAs using EVs into oomycete pathogen *Phytophthora capsici* to induce cross-kingdom RNAi^[43]^.

EV-mediated sRNA trafficking is also observed between mammalian hosts and their pathogens and parasites, demonstrating the important role of EVs in cross-organismal sRNA trafficking. In the aforementioned interaction between the nematode *H. polygyrus* and mice, *H. polygyrus* uses EVs to transport miRNAs into the mouse cells^[42]^. Similarly, human monocyte cells that deliver miRNAs into fungal pathogen *C. albicans* also rely on EVs for this cross-kingdom RNAi^[122]^. Within intra-organismal cellular interactions in mammalian systems, EVs have been shown to traffic functional mRNA transcripts between cells for protein translation in destination cells^[128,129]^. Human and mouse white blood cells produce EVs containing hundreds of functional mRNA species^[128]^. mRNA trafficking was a novel discovery in mammalian cell-cell communication and revealed a new function of EV trafficking^[127]^. Despite mRNA intra-organismal trafficking via EVs, there have been no reports to date of functional mRNA cross-kingdom trafficking in mammals, plants, or microbes.

## SELECTIVE LOADING OF SMALL RNAs INTO EVs

Selective sorting of sRNAs into EVs has been previously reported and continues to be characterized in many mammalian cell types. In human lymphocytes, hnRNPA2B1 protein was found to bind to sRNAs and load sRNAs into exosomes depending on specific sequence motifs^[130]^. A cleaved form of Rab-interacting lysosomal protein (RILP) also contributes to exosomal miRNA loading depending on a conserved RNA motif (AAUGC) in cultured HeLa cells^[131]^. Another study in mouse neurons showed that miRNAs that share a conserved four-nucleotide motif (GUAC) were selectively loaded into EVs^[132]^.

Although selective RNA loading into EVs based on conserved motifs or RNA-binding proteins is established in mammalian vesicle biology, research on selective loading is just beginning to be explored in the study of plant and microbial EVs. In Arabidopsis, EV-sRNA profiling analysis revealed that the enrichment of sRNA species in EVs does not correlate with their abundance inside the plant cell^[56]^, which suggests that these EV-enriched sRNAs are selectively loaded into EVs for transport. Proteomics analysis on purified EVs isolated from *B. cinerea* infected Arabidopsis leaves revealed several RNA-binding proteins, including Arabidopsis AGO1, DEAD-box RNA helicases (RH) 11, RH37, and RH52, which contribute to EV selective sRNA loading^[56]^. These RNA-binding proteins are localized in the membrane fractions inside the cell and selectively bind with a subset of sRNAs that are 20-22 nt long, with the first 5’ nucleotide being uracil. These RNA-binding proteins subsequently enter MVBs with associated sRNAs and are released within exosomes into the apoplast. Another set of EV-localized RNA-binding proteins Annexin (ANN) 1 and ANN 2 bind sRNAs non-specifically. Furthermore, *ago1*, *rh11/37,* and *ann1/2* mutants have reduced levels of EV-enriched sRNAs in EVs^[56]^. These results demonstrate that AGO1 and RH11/37/52 are important for selective sRNA loading into EVs, and that although ANN1/2 are not involved in the selective loading process, they are important for sRNA stabilization in EVs [Figure 1]^[56]^. Similar EV-mediated cross-species RNAi and RNA loading mechanisms exist in mammalian-parasite interactions. EVs of nematode parasite *H. polygyrus,* which mediate cross-species RNAi within its mammalian host^[42]^, contain an AGO protein (exWAGO) that specifically binds to EV-enriched RNAs^[133]^. Taken together, these results indicate that AGO proteins are one of the components that are mainly responsible for selective RNA sorting into EVs in plants and nematodes.

## PATHOGEN-DERIVED EXTRACELLULAR VESICLES AND THEIR BIOLOGICAL FUNCTIONS

EV secretion is a conserved process in all eukaryotic and prokaryotic cells^[10,45]^. Research has begun to demonstrate the important role of microbial EVs in mediating the ability of bacteria and fungi to interact with their hosts. Microbes, such as Gram-positive and Gram-negative bacteria, archaea, and fungi, have all been shown to release EVs^[134,135]^. Gram-negative bacteria release 50 to 300 nm EVs by pinching off the bacterial outer membrane, generating outer membrane vesicles (OMVs)^[136-138]^. It has been debated how vesicles can be released or taken up by organisms with thicker cell walls compared to the ease of vesicle movement within mammalian cells lacking cell walls. A recent review highlighted the plasticity of the fungal cell wall, allowing for the passage of vesicles as large as 200 nm in and out of fungal cells, despite the original measurement of fungal cell wall pores being much smaller^[139,140]^. Recent proteomics data showed that fungal EVs are enriched with cell wall-modifying enzymes^[50,141]^, which could allow for movement of fungal EVs through the plant cell wall. This emphasizes the dynamic and flexible nature of the cell wall and provides information on how vesicles could move in and out of the cells of thicker-walled organisms. As well, EV membranes are malleable and have sufficient structural plasticity to facilitate passing through the cell walls.

Microbial EVs contain specific cargoes, such as DNA, RNA, metabolites, and proteins, that could be involved in pathogenesis and modulation of host immunity^[135,137,141,142]^. Most microbial EVs are isolated from cellular cultures by differential centrifugation at a predetermined time point to avoid capturing dying cells^[134,141,143]^. Bacteria in the human gut have been shown to release bacterial EVs that can enter into the bloodstream and lymphatic system and travel to distant organs throughout the body^[144]^. Bacterial EVs can have beneficial or detrimental effects on human cells, such as inducing dendritic cell polarization or stimulating cancer proliferation^[144]^. The human fungal pathogen *Cryptococcus neoformans* also produces EVs that contain components of its fungal capsule, a cellular structure essential for virulence^[145]^. Proteomics analysis of EVs from five species within the genus *Candida* identified 36 common proteins enriched for orthologs of biofilm mediators of the mammalian pathogen *C. albicans*^[146]^. This study also discovered that vesicles from one *Candida* species can confer function to other *Candida* species, suggesting an important role of EVs in the development of microbial biofilm communities^[146]^. Scanning electron microscopy and cryo-transmission electron microscopy allowed the observation of EVs with an average size of 100 nm on the surface of the *Candida* biofilm, corroborating that EVs can easily move across the fungal cell wall^[146]^.

Over the past decade, studies of microbial EVs have mainly focused on mammalian-infecting pathogens, with limited reports on EVs derived from plant pathogens. To date, most of the plant-interacting bacteria from which EVs were isolated are Gram-negative bacteria, including *Xanthomonadaceae* family bacteria^[147-150]^, *Xylella fastidiosa*^[151,152]^, and *Pseudomonas syringae*^[153-155]^. Proteomic analysis of Gram-negative bacterial EVs reveals virulence factors and signaling molecule contents that may play a role in plant infection^[143]^. Direct evidence that phytopathogen-derived EVs contribute to virulence was provided by the study of the xylem-colonizing plant pathogenic bacterium, *X. fastidiosa*^[152]^. *X. fastidiosa*-derived EVs block *X. fastidiosa* cells from interacting with the xylem wall, which increases its systemic spread within the plant host and promotes virulence^[152]^. It has also been observed that animal pathogenic bacteria produce EVs that fuse with the host plasma membrane^[156]^. The opportunistic human pathogen *Pseudomonas aeruginosa* produces EVs that fuse with the host cell plasma membrane at lipid raft microdomains for long-distance delivery of bacterial virulence factors^[157]^. Similarly, the phytopathogen *Xanthomonas campestris* generates EVs that fuse directly with the Arabidopsis plasma membrane. This process depends on the clustering of the plant lipid raft proteins remorin 1.2 and remorin 1.3^[150]^. The observation that phytobacterial EVs fuse with the host plasma membrane suggests that EVs facilitate cross-kingdom delivery of pathogen cargo to plant cells. However, whether phytobacterial EVs also contain RNA or DNA molecules is still unknown. As indicated above, symbiotic Rhizobium bacteria transport tRFs into host root cells to silence host nodulation genes, suggesting that bacterial tRNA may act as an sRNA source to mediate cross-kingdom RNAi^[113]^. It is interesting to pose whether Rhizobium RNA trafficking is mediated by EVs [Figure 1].

More than twenty species of yeast and ﬁlamentous fungi have been observed to secrete EVs^[141]^. Fungal EVs are thought to be derived from MVBs or to bud directly from the plasma membrane^[135,142]^. In the phytopathogenic fungus *Ustilago maydis*, five effectors and two membrane proteins form a stable protein complex anchored in EV-like structures that may be derived from MVBs^[158]^. In plant pathosystems, EVs have been isolated from several filamentous fungi, including *Fusarium oxysporum*^[51]^, *Fusarium graminearum*^[49]^, wheat pathogen *Zymoseptoria tritici*^[50]^, corn smut fungus *Ustilago maydis*^[159]^, anthracnose pathogen *Colletotrichum higginsianum*^[160]^, post-harvest rot pathogen *Penicillium digitatum*^[47]^, and powdery mildew fungus *Blumeria hordei*^[99]^. These EVs were purified from culture supernatants or fungi grown on plant tissue and contained proteins, nucleic acids, lipids, metabolites, and polysaccharides. Proteomic analysis of *F. oxysporum* EVs detected functional proteins involved in secondary toxin metabolism, cell wall degradation, and protein degradation^[51]^. Furthermore, injection of *F. oxysporum* EVs into the leaves of cotton or *N. benthamiana* plants induced a phytotoxic response in plants, suggesting that fungal EVs likely play an important role in infection^[51]^. In addition, critical virulence-related proteins and effectors have been identified in fungal EVs. For instance, proteomic analysis of *F. graminearum* EVs identified protein effectors, some of which do not have predicted secretion signal peptides^[49]^. This suggests that EVs are an unconventional secretion pathway for fungal effectors. EVs isolated from the fungus *Z. tritici* also contain a number of putative virulence-associated proteins that may play a role in the infection of wheat^[50]^. Current phytopathogen effector analysis focuses exclusively on predicted secreted proteins with signal peptides. Proteomics analysis of pathogen EVs may help identify a novel class of previously unidentified pathogen effectors.

Studies of RNA in EVs from plant pathogenic fungi are limited. EVs from U. maydis contain mRNAs encoding metabolic enzymes, known effectors, and virulence protein^s[159]^. EVs isolated from the infection site of *Blumeria hordei* on barley plants were enriched in *B. hordei*-derived milRNAs, which have a potential role in host gene silencing^[99]^. As mentioned above, the fungal pathogens *B. cinerea*, *V. dahliae,* and the oomycete pathogen *H. arabidopsidis* have been shown to deliver sRNAs into plant host cells^[88,91,98]^. The mechanism of the delivery of these sRNAs is likely via fungal EVs [Figure 1], and many experiments have been initiated to test this hypothesis. Aside from RNA and proteins, one study found that *P. digitatum* uses EVs to transport phytotoxic metabolites into citrus fruit cells during infection^[47]^. This is one of the first characterized reports of cross-kingdom metabolite trafficking in a host-pathogen interaction, demonstrating the functional diversity of EVs and their potential to traffic a variety of biological cargoes that have yet to be discovered.

## POTENTIAL APPLICATIONS OF PLANT EVS

**Figure 3:**
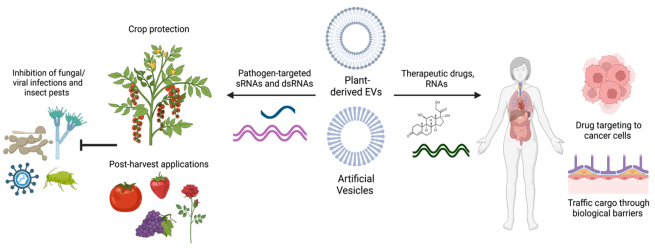
Potential Application for Plant EVs. Plant-derived EVs and artificial vesicles have been developed for agricultural crop protection and advances in human medicine. Artificial vesicles have been used to load and stabilize pathogen and pest-targeted sRNAs on plants^[163]^, as well as being used in drug delivery mechanisms for human medicine^[77,78]^. Plant-derived EVs have been explored for their native anticancer, anti-inflammatory, and other medicinal uses in humans, as well as their potential to uptake and deliver drugs through biological barriers within the body. This figure was created with https://www.biorender.com/.

The significant role of plant EVs in transporting genetic cargo in plant-pathogen interactions has motivated efforts to develop nanocarrier mimics of these natural EVs for RNAi-based pathogen control strategies in agriculture and as drug delivery agents in the medical field [Figure 3]. These organic nanocarriers can consist of various materials, including lipid-based artificial nanovesicles (AVs) and plant-derived nanovesicles (PDNVs). AVs have recently been used for dsRNA delivery in spray-induced gene silencing (SIGS) to improve the stability of dsRNA in the environment^[39,161-163]^. SIGS is a powerful and eco-friendly method for crop protection. Many pathogens can efficiently take up RNAs from the environment^[88,163]^, which makes SIGS possible. Topical application of exogenous RNA targeting pest/pathogen virulence genes results in gene silencing and subsequent disease inhibition. AVs formulated with 1,2-dioleoyl 3-trimethylammonium-propane (DOTAP) + polyethylene glycol (PEG), 1,2-dioleyloxy-3-dimethylaminopropane (DODMA), or DOTAP alone were found to provide strong and prolonged protection of dsRNA against *B. cinerea* on pre- and post-harvest materials^[163]^. Encapsulation of dsRNA in AVs extended RNAi-based protection for up to 10 days on fruit and 21 days on grape leaves^[163]^. Similarly, other reports have investigated the use of liposomes and lipid transfection reagents for SIGS against insect pests^[164,165]^.

As mentioned above, PDNVs from various fruit and vegetable sources have attracted considerable attention for nanomedicine and drug delivery due to their many favorable properties [Figure 3]. Since PDNVs are derived from plants, they are biocompatible, biodegradable, and easy to scale up. In addition, the small size of PDNVs enhances cellular uptake in humans. PDNVs can also tolerate the gastrointestinal tract and cross biological barriers such as the blood-brain barrier to deliver biological molecules^[166,167]^. PDNVs may also have unique inherent properties depending on the plant source. For example, PDNVs derived from lemon juice exhibited anticancer activity^[168]^, while ginger EVs possessed anti-inflammatory activity and were preferentially taken up by microbes^[75,169]^. As well, watermelon-derived extracellular vesicles can influence intestinal cell secretions and consequently, the placental proteome^[170]^. Thus, PDNVs are gaining interest not only as nanocarriers but also as therapeutic agents themselves that can modulate the activity of microbial and mammalian cells. This has led to significant efforts to characterize the cargo and composition of these PDNVs from various juice sources to better understand their biological functions.

Modifications of PDNVs can make them more suitable for use in therapeutics. For example, lipids can be extracted from PDNVs and reassembled into nanoparticles or treated with chemicals to modify their structure and properties. These types of modifications have been shown to make PDNVs more amendable to the loading of RNAs or specific drugs^[171]^. More work is needed to fully understand the depth and breadth of PDNVs^[166,167]^. Finally, PDNVs could have significant agricultural applications by delivering biological cargoes in pathogen control strategies. In particular, the environmentally friendly and scalable nature of PDNVs makes them very attractive compared to current synthetic nanocarriers.

## DISCUSSION

The recent emergence and expansion of the studies of plant and microbial EVs has demonstrated the powerful role of EVs in intercellular communication between organisms, taking the field of vesicle biology a new step further beyond the realm of intra-organismal cellular communication. There are evident gaps in the knowledge around plant and microbial EV cargo, biogenesis, and their role in RNA, protein, and metabolite trafficking. New insights into EV-mediated cross-kingdom trafficking are emerging in several host-pathogen/symbiont interactions in both the plant and mammalian fields, with the potential for many more discoveries between species. As we have highlighted, cross-kingdom RNAi is bidirectional, and sRNA trafficking between plants and their interacting organisms induces gene silencing *in trans*. Cross-kingdom RNAi exists in plant-fungal interactions, as well as between plants and other microbes and pests, including bacteria, oomycetes, and parasites. While nematodes, bacteria, and yeast have been shown to traffic biological material into their mammalian hosts^[42,141,143,144]^, recent studies have shown that mammalian hosts also use EVs to traffic sRNAs to interacting organisms^[122]^.

There is clear evidence that plants use EVs to transport RNA and proteins into interacting organisms, analogous to how mammalian cells use EVs to shuttle RNA and other biological materials between cells or tissues within a single organism or between interacting organisms. Similarly, microbes generate EVs with a variety of contents in order to communicate with other microbes or their hosts. The study of EVs is expanding our understanding of organismal interactions. A new study by Hackl *et al.* discovered that the marine bacterial species *Prochlorococcus* secretes vesicles containing strain-specific DNA transposons that facilitate horizontal gene transfer of metabolic and bacteriophage resistance genes between *Prochlorococcus* strains^[172]^, demonstrating that these EVs are very stable and can survive in seawater. Recently, it was also shown that the algal species *Emiliania huxleyi*, when infected with *E. huxleyi* virus, secretes vesicles containing sRNAs that influence the population dynamics of interacting marine microorganisms such as phytoplankton and bacteria^[173]^. These studies demonstrate the essential role of vesicles in the assembly and communication of marine microbial communities, highlighting the underappreciated role of EVs in inter-organismal cellular interactions, microbial evolution, and environmental stability. More research is needed to understand how microbes such as bacteria and fungi use EVs for adaptation and intra- and inter-species communication.

Although five distinct classes of plant EVs have been identified, the characterization of their biological functions, cargoes, biogenesis and biomarkers lags behind that of mammalian EVs. Furthermore, studies of microbial EVs are even further limited. The functions of cargoes between species are often not as apparent as those between tissues within the same organism. Thus, the cargoes and biological functions of EVs in plants and microbes are and will continue to be an intriguing yet challenging direction in the field of vesicle biology. The current task of the field is to identify more EV markers in plants and microbial species and to improve methods to isolate EVs and purify specific classes of EVs.

Although EV-mediated transport is a key mechanism for RNA protection and delivery between hosts and microbes, EV-independent RNA transport has also been reported to date. In human plasma, extracellular RNA and protein complexes were identified, such as AGO protein-RNA complexes and high-density lipoprotein-RNA complexes^[174,175]^. In plants, nearly 30% of host Arabidopsis sRNAs found in *B. cinerea* cells were not found in EVs^[29]^, suggesting an EV-independent pathway for transporting these sRNAs. However, EV-independent extracellular RNAs and RNA-protein complexes would likely undergo rapid degradation in the plant extracellular environment, which contains numerous nucleases and proteases^[53,54]^. It remains to be explored how EV-independent extracellular RNAs and RNA complexes are secreted and survive in extracellular environments, and whether these RNAs are functional and mediate cross-kingdom RNAi. Overall, encapsulation of RNA in EVs is an effective strategy for cells to protect extracellular RNA from degradation and may also mediate efficient RNA uptake into interacting cells and organisms.

A better understanding of plant EVs and cross-kingdom RNAi will promote the development and application of a new generation of RNA “fungicides” to control plant diseases caused by eukaryotic pathogens^[35,163]^. In addition, plant-derived EVs and nanovesicles have a potential role in novel medical therapies^[77,78]^. Research has begun to develop PDNVs into therapeutic agents because of their inherent ability to interact with human cells and influence protein and metabolite composition. However, more research is required to fully characterize the diversity of cargoes and the overall effects of PDNVs. In the future, plant-derived EVs could be engineered to carry specific cargoes, such as therapeutic RNAs or drugs, while providing a protective envelope and effective delivery of the therapeutic agents into human cells.

Gradually, research on plant and microbial EVs will begin to catch up with the greater knowledge of mammalian EVs, and the field of EV-mediated intra-organismal cell-to-cell communication and cross-kingdom/organismal communication will continue to grow. No cell exists alone, and whether as part of the tissues of a larger organism or as a single-celled member of a microbial community, research is increasingly demonstrating the importance of EVs in mediating cargo exchange and communication between cells, tissues, and organisms.

## DECLARATIONS

### Copyright

© The Author(s) 2023.
